# Liver-specific loss of *Atg9a* perturbs lipid metabolism and hepatocyte integrity

**DOI:** 10.1080/27694127.2025.2551028

**Published:** 2025-09-02

**Authors:** Elodie Mailler, Asmita Singh, Michal Jarnik, Yan Li, Lynne Holtzclaw, Victoria Hoffmann, Sohtaro Mine, Paulina Stallcup, Laleh Ordoubadinia, Carlos M. Guardia

**Affiliations:** a Division of Neurosciences & Cellular Structure, Eunice Kennedy Shriver National Institute of Child Health and Human Development, National Institutes of Health, Bethesda, MD, USA; b Placental Cell Biology Group, National Institute of Environmental Health Sciences, National Institutes of Health, Durham, NC, USA; c Proteomics Core Facility, National Institute of Neurological Disorders and Stroke, National Institutes of Health, Bethesda, MD, USA; d Microscopy and Imaging Core, Eunice Kennedy Shriver National Institute of Child Health and Human Development, National Institutes of Health, Bethesda, MD, USA; e Division of Veterinary Resources, Office of the Director, National Institutes of Health, Bethesda, MD, USA; f Medical Virology Section, Laboratory of Infectious Diseases, National Institute of Allergy and Infectious Diseases, National Institutes of Health, Bethesda, MD, USA; g Department of Laboratory Medicine, Clinical Center, National Institutes of Health, Bethesda, MD, USA

**Keywords:** ATG9A, autophagy, lipid droplets, liver, mitochondria, mouse

## Abstract

The autophagy-related protein ATG9A is integral to cellular autophagy and lipid mobilization, yet its importance in mammalian physiology remains underexplored. Using a liver-specific conditional *Atg9a* knockout (*Atg9a*-cKO) mouse model, we uncovered critical insights into the physiological function of ATG9A in this organ. *Atg9a*-cKO mice exhibited hepatomegaly, abnormal hepatocyte morphology, mitochondrial fragmentation, and lipid droplet accumulation. Blood chemistry and proteomics analyses revealed elevated serum cholesterol, reduced albumin, and dysregulation of pathways related to lipid metabolism and oxidative stress responses. These findings establish an essential role for ATG9A in maintaining hepatocyte integrity, lipid trafficking, and overall liver health, offering a model for studying autophagy-related hepatic pathologies.

## Introduction

Macroautophagy (commonly referred to as autophagy) is a catabolic process for the lysosomal degradation of cytoplasmic components in all eukaryotic cells. Autophagy is carried out by the hierarchical function of several autophagy-related (ATG) proteins and is essential for the response to cellular stress triggered by starvation, hypoxia, infection, oxidative stress, and inflammation [1]. Autophagy requires the formation of a double-membrane organelle, the autophagosome, which expands while engulfing bulk cytoplasmic components (nonspecific) or selected cytoplasmic cargos (specific) [2]. Selective autophagy is mediated by cargo receptors that recruit cargo to the inner membrane of the phagophore [3]. Ultimately, the mature autophagosome fuses with endolysosomes, leading to the degradation and recycling of the luminal content back into the cytoplasm for their reuse by the cell. Defects in any of these autophagy steps are associated with a myriad of diseases, including cancer and neurodegeneration [4].

One of the indispensable ATG gene products is ATG9A, the only transmembrane protein of the core autophagy machinery. ATG9A is ubiquitously expressed, unlike an ATG9B paralog that is mostly enriched in the placenta and pituitary gland [5]. At the molecular level, both ATG9 proteins function as lipid scramblases [6–8] to distribute phospholipids between the two leaflets of the membranes into which they are inserted. This activity is thought to provide a way to maintain membrane integrity, while autophagosomes expand by ATG2-mediated transfer of lipids from other membrane sources [9]. Knowledge of the ATG9 transmembrane domain topology [6,7,10] has recently suggested a mechanism for its phospholipid-scrambling activity. ATG9 homologs form a homotrimer with a central pore and lateral channels that connect the cytoplasmic leaflet of the membrane with the central cavity. These cavities are responsible for providing access, accommodating, and flipping the polar head of phospholipids, while the trimer undergoes open-close conformational changes. Although these structural studies provided unique insights about the molecular function of ATG9 proteins, there is neither experimental support for such mechanism nor an explanation of how the flux of lipids is controlled in the context of the cell and a whole organism. Remarkably, ATG9A has been implicated in cellular processes other than autophagy, such as cell migration [11], plasma membrane repair [12], dsDNA-induced immune response [13], and lipid mobilization [14]. However, it remains to be determined whether the role of ATG9A in these processes is direct, stems from impaired autophagosome maturation, or reflects an indirect involvement of autophagy in a context-dependent fashion.

Even more limited are *in vivo* studies of ATG9A in higher mammals. The first report of an *Atg9a* null mouse described phenotypes similar to those seen in *Atg5-*, *Atg7-*, and *Atg16l1*-deficient mice, with death occurring within one day of delivery [13]. Subsequent work showed severe growth restriction in *Atg9a*^−/−^ fetuses, with an increased likelihood of *in utero* death and resorption [15], explaining the lower-than-expected Mendelian frequency in the offspring. However, the mice used in these studies had a hybrid background. When similar studies were performed in a C57BL/6J background, all *Atg9a* null mice died around embryonic day 15 [16]. Central nervous system-specific *Atg9a* conditional knockout (cKO) mice showed growth retardation, severe degenerative neurological phenotypes, and half died within the first week after delivery, while the remaining died by 4 weeks of age [16]. Beyond the heterosis shown in these early studies, the reasons for the heterogeneous outcomes associated with the absence of ATG9A remain unclear. In humans, only four studies have found *ATG9A* variants or association with disease to date [17–20], two involving defects in ovarian biology and the other two in immune system dysfunction.

To further investigate the physiological roles of ATG9A, we characterized a mouse conditionally deficient in *Atg9a* in the liver using a Cre-lox approach. Our study showed that live-specific *Atg9a*-cKO mice were viable but displayed enlarged livers, with altered tissue architecture, autophagic defects, fragmented mitochondria, and lipid droplet accumulation in hepatocytes. Moreover, blood chemistry and proteomics revealed signs of liver damage, inflammation, and misregulation of lipid homeostasis. Overall, the hepatocytes from these mice exhibited similar phenotypes to those cells with knockout (KO) of ATG9A, demonstrating the physiologically relevant role of ATG9A in lipid mobilization in hepatocytes.

## Results

### Liver-specific Atg9a KO mice exhibit pronounced hepatomegaly

**Figure 1.**
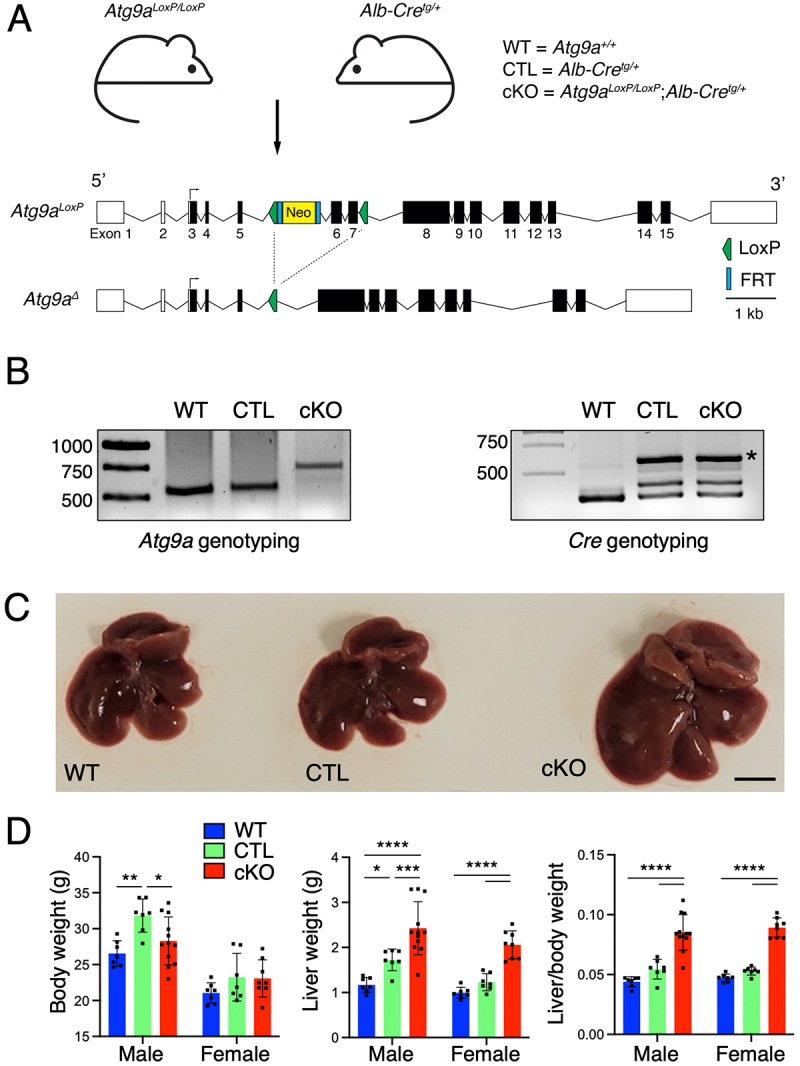
*Atg9a* deficiency in hepatocytes causes hepatic hypertrophy in mice.

To further examine the physiological role of ATG9A, we generated KO mice for *Atg9a* specifically in the liver using the Cre-lox system in C57BL/6 mice. Homozygous floxed *Atg9a* (*Atg9a*^*LoxP/LoxP*^) mice were crossed with *Alb-Cre* (*Alb-Cre*^*tg/+*^) mice to produce *Atg9a*-cKO mice (*Atg9a*^*LoxP/LoxP*^;* Alb-Cre*^*tg/+*^) (Figure 1A). Wild type (WT) and *Alb-Cre* mice (CTL) were used as controls in subsequent experiments. Genotyping for WT, LoxP, and Cre alleles was done by PCR analysis (Figure 1B). Although *Atg9a* null mice are embryonically lethal in this genetic background [16], *Atg9a*-cKO animals were born at the expected Mendelian ratio (24 ± 16% [standard deviation] vs. 25% theoretical) and survived until tissue collection time (up to 3 months). However, *Atg9a*-cKO mice exhibited pronounced hepatomegaly (Figure 1C) in comparison to WT or CTL mice, as evidenced by both the absolute weight of their livers and the ratio of liver to total body weight, regardless of sex (Figure 1D). These results demonstrated a dramatic increase in liver size upon depletion of *Atg9a*, consistent with what has been shown for *Atg5*, *Atg7,* and other KO livers [21–24].

A. Schematic representation of breeding scheme for generating *Atg9a*-cKO mice with *Cre/Lox* tissue-specific system. B. PCR analyses of genomic DNA extracted from wild-type control (WT), heterozygous *Alb-Cre* control (CTL), and *Atg9a*-cKO (cKO) mice tissues. The positions of molecular mass markers (in bp) are indicated on the left of each panel. C. Eleven-to-thirteen-week-old *Atg9a*-cKO mice and their counterpart control littermates were sacrificed. Representative pictures of their livers are shown (scale bar = 1 cm). D. Body and liver weights were measured for both males and females (each dot represents a single animal). The liver/body weight ratio was also calculated. Bar graphs represent the mean ± SD of these values. Statistical significance was determined using two-way ANOVA with Tukey post-hoc test (*p < 0.05, **p < 0.01, ***p < 0.001, ****p < 0.0001).

### Hepatocyte alterations in Atg9a-cKO mice

**Figure 2.**
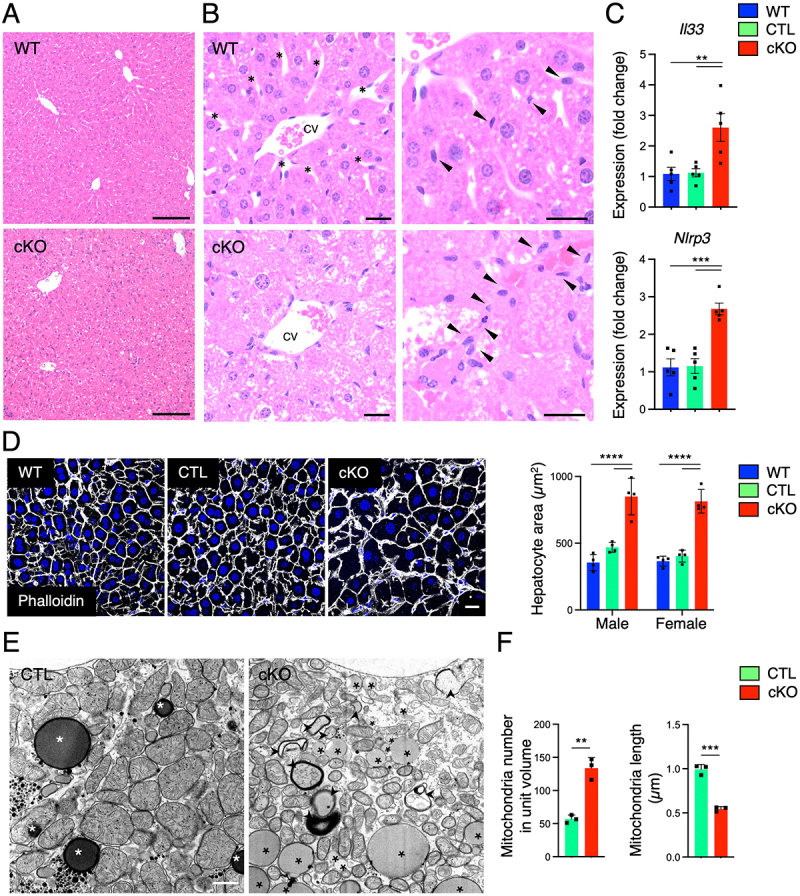
Histological findings of the liver in *Atg9a*-cKO mice.

In light of the striking liver phenotype observed in the *Atg9a*-cKO mice, we decided to investigate in more detail the histology of this tissue. Examination of formalin-fixed, paraffin-embedded (FFPE) livers of *Atg9a*-cKO mice revealed significant histological changes indicative of hepatocyte dysfunction (Figure 2). The loss of *Atg9a* led to diffuse cytomegaly of hepatocytes with increased, variably clear staining cytoplasm (Figure 2A), as well as loss of the characteristic central location of nuclei of healthy symmetric polygonal hepatocytes (Figure 2B). Additionally, sections of *Atg9a*-cKO tissue showed disruption of sinusoidal spaces (Figure 2B, asterisks) and hypertrophy of cells lining the sinusoids (Figure 2B, arrows). Analysis of inflammatory markers such as *Il33* and *Nrlp3* [25–27] by RT-qPCR showed a significant upregulation of transcripts in the cKO tissue (Figure 2C). Staining of cortical actin filaments with phalloidin highlighted the severe change in morphology of hepatocytes (Figure 2D), where cells almost doubled in size in comparison to controls (Figure 2D). Transmission electron microscopy performed on perfused animals (Figure 2E), showed a significant increase in the number of mitochondria with a concomitant reduction in their size (Figure 2F). Fragmentation of mitochondria in hepatocytes has been associated with liver damage and impaired energy production [28]. Given the lack of increased proliferation or apoptosis in *Atg9a*-cKO livers when compared to controls (Supplementary Figure S1), the hepatomegaly phenotype might be mainly explained by hepatocyte cytomegaly.

A. Representative images of haematoxylin and eosin (H&E) staining of WT and *Atg9a*-cKO liver sections (representative pictures from females). Notice the cytomegaly, including loss of characteristic nuclear-to-cytoplasm symmetry and the obscuring of hepatic cord and sinusoid architecture in the *Atg9a*-cKO liver. Scale bars: 200 μm. B. Representative images of H&E staining of WT and *Atg9a*-cKO liver sections (representative pictures from females) at higher magnification. Asterisks show normal, open sinusoid spaces in WT tissue (which are obscured by enlarged hepatocytes in the cKO), while arrows highlight sinusoidal lining cells. Notice the disrupted sinusoids in *Atg9a*-cKO livers. CV, central vein. Scale bars: 20 μm (*n* = 3). C. Selected markers of inflammation were analyzed by RT-qPCR using RNA extracted from FFPE sections of WT, CTL, and *Atg9a*-cKO livers. Given that female and male samples showed similar trends, both sexes were combined to increase the number of biological replicates and reach statistical power. Each dot represents a single animal. Statistical significance was determined using one-way ANOVA with Tukey post-hoc test (**p<0.01, ***p<0.001). D. Confocal fluorescence microscopy of WT, CTL, and *Atg9a*-cKO frozen liver sections that were stained for actin with phalloidin (gray). Nuclei were stained with DAPI (blue). Scale bar: 20 μm. Results are representative of four animals per sex and genotype. The area of each cell was quantified in 25–29 cells for *Atg9a*-cKO samples and 37–43 cells for each control using Image J. Bar graphs represent the mean ± SD fold-change of these values. Statistical significance was determined using two-way ANOVA with Tukey post-hoc test (****p < 0.0001). E. Electron micrographs of CTL and cKO mouse hepatocytes in primary culture. Results are representative of three animals per genotype. Asterisks indicate lipid droplets and arrowheads are aberrant autophagosomes. Scale bar: 1 μm. F. The number and size of mitochondria were quantified in CTL and *Atg9a*-cKO hepatocytes using Image J. Results are representative of three animals per genotype. Bar graphs represent the mean ± SD of these values. Statistical significance was determined using the Student's t-test (**p < 0.01, ***p < 0.001).

### Autophagy deficiency in Atg9a-cKO liver

**Figure 3.**
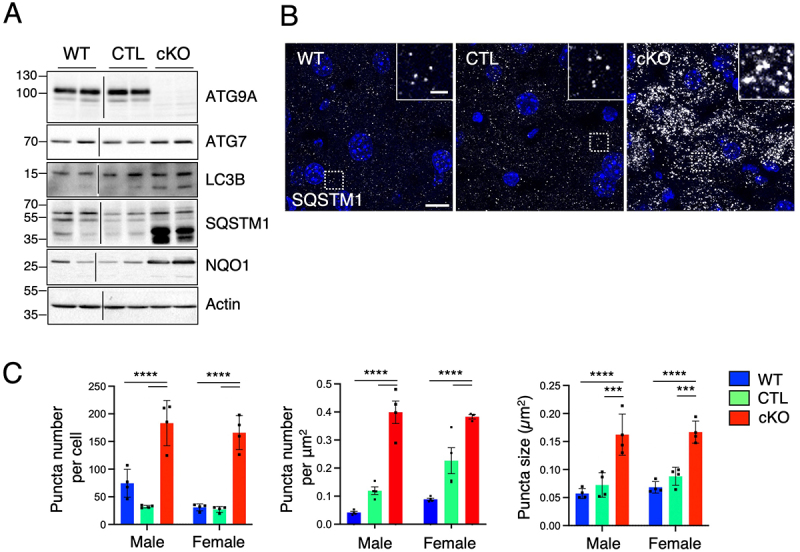
*Atg9a* deficiency in the liver impairs autophagy.

To assess autophagy function in the livers from *Atg9a*-cKO mice, we performed whole-tissue immunoblotting for the autophagy proteins ATG9A and ATG7, together with the autophagosome marker LC3B and cargo receptor SQSTM1/p62 (Figure 3A). The absence of ATG9A in the cKO lanes confirmed the depletion of ATG9A at the protein level. No significant changes were observed in the levels of ATG7. As previously shown in *ATG9A* KO cells [29], we observed a significant increase of LC3B-II and both full-length and shorter forms of SQSTM1 in the cKO liver (Figure 3A) that translated into a ~ threefold increase in both size and number of SQSTM1-positive puncta when examined using immunohistochemical analysis of frozen sections from these livers (Figure 3B,C). In line with previous studies showing that the KO of *Atg5* [21] or *Atg7* [30] caused an increase in the levels of NAD(P)H:quinone oxidoreductase (NQO1), our *Atg9a*-cKO mice similarly exhibited a significant increase in NQO1, consistent with activation of Nrf2-NQO1-mediated liver detoxification pathways [31,32].

A. SDS-PAGE and immunoblot analysis of WT, CTL, and *Atg9a*-cKO lysates from liver tissues using antibodies to the proteins indicated on the right of each panel. Actin was used as a loading control. The positions of molecular mass markers (in kDa) are indicated on the left of each panel. Both male (odd lanes) and female (even lanes) liver tissue was loaded for each genotype. Results are representative from three independent experiments. B. Confocal fluorescence microscopy of WT, CTL, and *Atg9a*-cKO frozen liver sections that were stained with antibody to SQSTM1 (gray). Nuclei were stained with DAPI (blue). Scale bar: 10 μm. Results are representative of four animals per sex and genotype. Insets show enlarged views of the boxed areas. Scale bar: 2 μm. C. The number per cell and per area and size of puncta were quantified in 30 cells per animal in four animals per sex and genotype using the ‘Analyze particles’ function of Image J. Bar graphs represent the mean ± SD of these values. Statistical significance was determined using two-way ANOVA with Tukey post-hoc test (***p < 0.001, ****p < 0.0001).

**Figure 4.**
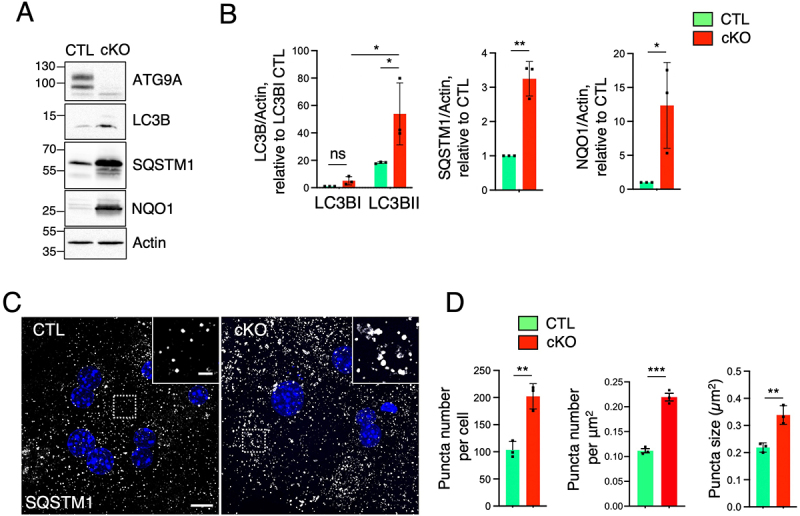
Defective autophagy in isolated hepatocytes from *Atg9a*-cKO mice.

Similar phenotypes were observed in hepatocytes isolated from the rest of the tissue and cultured *in vitro* (Figure 4). In addition to the increased levels of LC3B-II and NQO1, full-length SQSTM1 buildup was much more evident (Figure 4A,B) than in the whole-tissue lysates (Figure 3A). Immunofluorescence microscopy experiments demonstrated a twofold increase in the number and size of cytosolic SQSTM1 aggregates in the *Atg9a*-cKO hepatocytes (Figure 4C,D). Altogether, these results demonstrated that the phenotype observed in the tissue were likely the result of changes in the hepatocytes.

A. SDS-PAGE and immunoblot (IB) analysis of CTL and *Atg9a*-cKO lysates from hepatocytes in primary culture using antibodies to the proteins indicated on the right of each panel. Actin was used as a loading control. The positions of molecular mass markers (in kDa) are indicated on the left of each panel. Results are representative from three independent experiments. B. Bar graphs represent the mean ± SD of LC3B, SQSTM1, and NQO1 levels normalized for actin and relative to CTL, from three independent experiments. Statistical significance was determined using the Student’s t test (ns *p*>0.05, *p < 0.1, **p < 0.01). C. Confocal fluorescence microscopy of CTL and *Atg9a*-cKO hepatocytes in primary culture that were stained with antibody to SQSTM1 (gray). Nuclei were stained with DAPI (blue). Scale bar: 10 μm. Results are representative of the three independent experiments. Insets show enlarged views of the boxed areas. Scale bar: 2 μm. D. The number of SQSTM1-positive puncta per cell and per area and their size is quantified in 22 cells per animal in three independent experiments using the ‘Analyze particles’ function of Image J. Bar graphs represent the mean ± SD of these values. Statistical significance was determined using the Student’s t test (**p < 0.01, ***p < 0.001).

### Accumulation of lipid droplets in Atg9a-deficient hepatocytes

**Figure 5.**
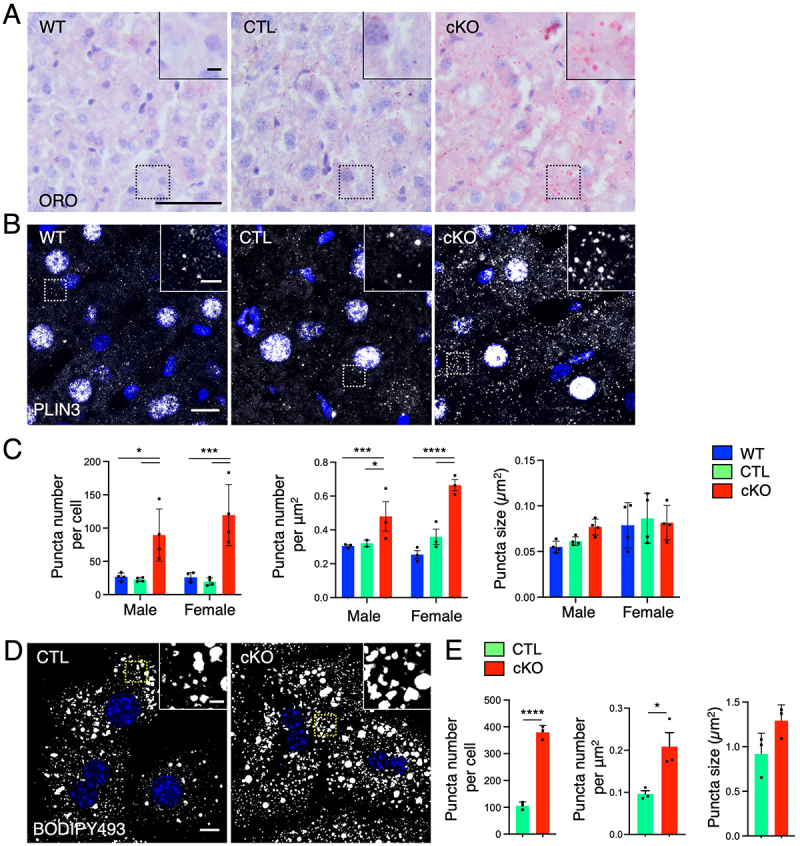
*Atg9a* deficiency in hepatocytes increases the number of lipids droplets.

In addition to the mitochondria alterations, the ultrastructural studies showed a clear increase in lipid droplets in *Atg9a*-cKO hepatocytes (Figure 2E, asterisks). This accumulation was confirmed by staining of thin-sliced sections with Oil-Red O (ORO), a lipophilic dye that labels neutral lipids, cholesteryl esters, and lipoproteins, and with antibody to PLIN3, a structural protein of lipid droplets (Figure 5A,B). Quantification of PLIN3 immunofluorescence showed a significant increase in the number of lipid droplets in the *Atg9a*-cKO tissue without a change in their size compared to controls (Figure 5C). Similar analysis done in isolated hepatocytes and using BODIPY as a fat-soluble dye to stain lipid droplets confirmed these results (Figure 5D,E). Our experiments thus demonstrated that *Atg9a*-deficient hepatocytes contain a significantly higher number of lipid droplets, suggesting that *Atg9a* is essential for regulating lipid storage within the liver.

A. Frozen liver tissues were subjected to Oil Red O (ORO) staining. Results are representative of one animal per sex and genotype. Scale bar: 50 μm. Insets show enlarged views of the boxed areas. Scale bar: 5 μm. B. Confocal fluorescence microscopy of WT, CTL, and *Atg9a*-cKO frozen liver sections that were stained with antibodies to PLIN3 (gray). Nuclei were stained with DAPI (blue). Scale bar: 10 μm. Results are representative of four animals per sex and genotype. Insets show enlarged views of the boxed areas. Scale bar: 2 μm. C. The number per cell and area and the size of lipid droplets were quantified in 30 cells per animal in four animals per sex and genotype using the ‘Analyze particles’ function of Image J. Bar graphs represent the mean ± SD of these values. Statistical significance was determined using two-way ANOVA with Tukey post-hoc test (*p < 0.05, ***p < 0.001, ****p < 0.0001). D. Confocal fluorescence microscopy of CTL and *Atg9a*-cKO hepatocytes in primary culture that were stained for lipid droplets with BODIPY 493 (gray). Nuclei were stained with DAPI (blue). Scale bar: 10 μm. Results are representative of the three independent experiments. Insets show enlarged views of the boxed areas. Scale bar: 2 μm. E. The number per cell and area and the size of lipid droplets were quantified in 23 cells per sample in three independent experiments using the ‘Analyze particles’ function of Image J. Bar graphs represent the mean ± SD of these values. Statistical significance was determined using the Student’s t test (*p < 0.05, ****p < 0.0001).

### Blood chemistry alterations in Atg9a-cKO mice

**Figure 6.**
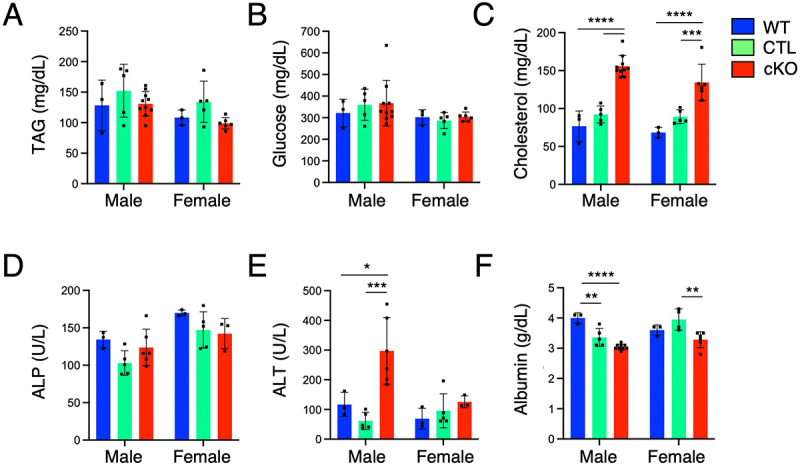
Biochemical analysis of serum from WT, CTL and *Atg9a*-cKO mice.

The increased size of the liver, defective autophagy, intracellular organelle changes, and activation of detoxification pathways indicated that the *Atg9a*-cKO animals may be under metabolic stress due to a malfunctioning liver. To corroborate a more systemic effect, we performed blood chemistry analysis from fed animals (Figure 6). This analysis revealed elevated levels of cholesterol (Figure 6C) and decrease in albumin concentration (Figure 6F) and a significant increase in alanine aminotransferase (ALT) levels only in males (Figure 6E). Triglycerides, glucose, and alkaline phosphatase (ALP) levels were similar to controls in *Atg9a*-cKO from both sexes (Figure 6A,B,D). These changes indicated mild early signs of liver damage.

Bar graphs represent the mean ± SD of each analyte measured. Sample size varies depending on the serum volume available from each mouse and the hemolysis index thresholds set (see Material and Methods). Each dot represents a single animal. Statistical significance was determined using two-way ANOVA with Tukey post-hoc test (*p < 0.05, **p < 0.01, ***p < 0.001, ****p < 0.0001). TAG: triglyceride; ALT: alanine aminotransferase; ALP: alkaline phosphatase.

### Proteomics changes in Atg9a-cKO livers

**Figure 7.**
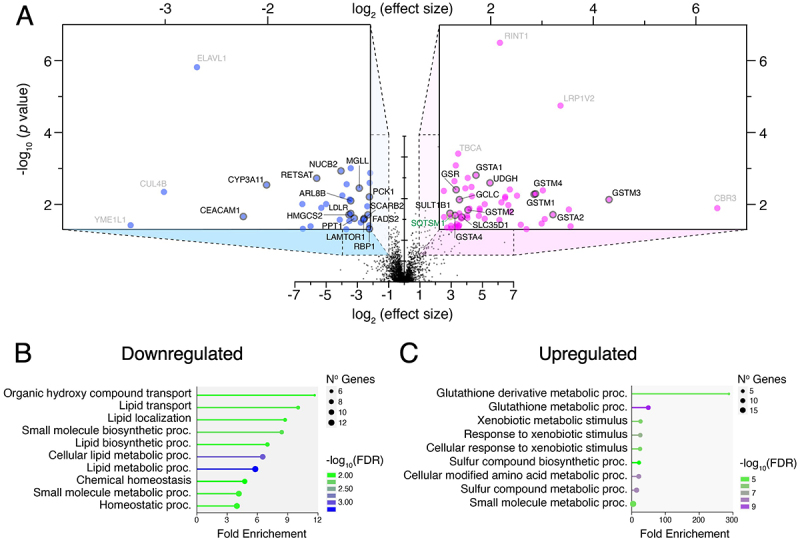
Proteomics analysis reveals disruption of lipid metabolism and oxidative stress pathways in liver from *Atg9a*-cKO mice.

Finally, we performed unbiased proteomics analysis in whole-liver lysates to assess differences between the *Atg9a*-cKO and control mice. The results were represented as a volcano plot (Figure 7A; Supplementary File 1) where thresholds of an effect size of at least two and a *p*-value smaller than 0.05 were applied and highlighted as relevant differentially expressed proteins. Additionally, all the downregulated (Figure 7B) and upregulated (Figure 7C) proteins from the volcano plot were examined together by gene ontology enrichment analysis [33]. In line with the above-mentioned observations, these experiments revealed significant alterations in protein expression related to lipid metabolism and oxidative stress (Figure 7B,C). Key downregulated pathways included those associated with lipid synthesis, transport, and storage, while pathways linked to glutathione metabolism and xenobiotic detoxification were upregulated (Figure 7B,C; Supplementary File 2).

A. Volcano plot showing *Atg9a*-cKO vs. CTL proteomics of whole liver lysates from four different mice (two females and two males) per genotype. Insets show proteins with significantly decreased levels (left, light blue; *p*<0.05 and effect size<-0.5) and increased levels (right, light magenta; *p*<0.05 and effect size >2) in *Atg9a*-cKO compared to CTL control livers. Select proteins are labeled (for a complete list, see Supplementary File 1). B,C. Top GO terms associated with proteins were significantly decreased (B) and increased (C) in the *Atg9a*-cKO vs. CTL mice.

## Discussion

This study highlights the pivotal role of ATG9A in hepatic autophagy and lipid metabolism. Using liver-specific *Atg9a*-KO mice, we demonstrated that the loss of ATG9A disrupts hepatocyte homeostasis, resulting in hepatomegaly, mitochondrial size changes, and lipid droplet accumulation. The autophagy defect was evidenced by increased LC3B-II and SQSTM1 levels (Figures 3A and 4A), as previously observed in human *ATG9A*-KO cell lines, suggesting the conjugation of LC3B-I to aberrant autophagosomes (Figure 2E) or protein aggregates in the cytosol [10,34–36]. The accumulation of SQSTM1/p62 has been shown to trigger the dissociation of Keap1 from Nrf2, leading to upregulation of cytoprotective genes and hepatic detoxification enzymes such as NQO1 [31,32], a phenomenon that we also observed in our *Atg9a*-liver KO mice (Figures 3A and 4A). Remarkably, in our study, we observed a dramatic accumulation of short SQSTM1/p62 isoforms in the cKO liver samples (Figure 3A), which are probably cleaved by caspase activation [37–39], signaling the early onset of liver toxicity [40]. While the absence of functional autophagosomes could explain most of the additional effects observed, how and when the Nrf2- and caspase-p62 axis are affected by *Atg9a* deficiency remains to be elucidated.

Sterile inflammation is a central driver of liver disease progression, including fibrosis [41,42], arising from crosstalk between immune and nonimmune cells that can either restore homeostasis or propel chronic inflammation and scarring [43]. Within this response, inflammasomes (particularly the NLRP3 complex) serve as key innate immune regulators [44]. NLRP3 activation by damage-associated molecular patterns proceeds through a two-step sequence: a priming phase that increases NLRP3, caspase-1, and pro-IL-1β transcripts, followed by posttranslational modifications that license the complex for activation [27,45]. In parallel, IL-33, an IL-1 family cytokine predominantly produced by stressed hepatocytes, is released upon cell lysis and functions as an alarmin that triggers repair programs and fibrogenesis [25,26,46,47]. Because both NLRP3 and IL-33 pathways are engaged in sterile inflammation-associated liver disease [48], we assessed their expression in *Atg9a-cKO* livers and found elevated inflammatory markers (Figure 2C) despite no overt fibrosis or apoptosis (Figure 2A,B, Supp. Fig. S1), consistent with an early inflammatory phase that may precede histologic remodeling. Whether this inflammatory priming is driven by autophagy defects, lipid accumulation, or their oxidation products [49] remains to be clarified. Future work should test causality and expanded profiling of complementary repair/fibrosis pathways to define how these axes jointly shape hepatic inflammation and liver disease [43,50].

The liver is central to metabolic regulation dependent on proper mitochondrial activity. The processes of mitochondrial fission and fusion, which govern mitochondrial dynamics, play a crucial role in shaping mitochondria and maintaining their functionality. Fusion helps preserve mitochondrial integrity by compensating for functional deficits and extending their lifespan. In contrast, fission isolates damaged mitochondria, facilitating their clearance through mitophagy, the selective degradation of damaged mitochondria by autophagy [51–53]. The presence of more and smaller mitochondria (Figure 2E,F) in the *Atg9a*-cKO cells is consistent with a perturbation of the normal fusion-fission dynamics but also autophagy deficiency (lack of clearance of mitochondria). Normal autophagy disruption by the lack of autophagosome formation in the *Atg9a*-cKO hepatocytes and the ensuing liver toxicity could damage mitochondria and upregulate systemic fission. The mitophagy defect would then result in accumulation of fragmented mitochondria. It remains unclear whether ATG9A could also have direct effects on mitochondria health given the previously observation of reduced fatty acid import and respiration rate of mitochondria in human *ATG9A*-KO cells [14]. Follow-up studies using the mKeima reporter mice [54] in an *Atg9a-cKO* background could help tackle deeper mitophagy phenotypes in this model.

Our *Atg9a*-cKO mice presented a remarkable accumulation of lipid droplets, which is consistent with recent insights on the involvement of ATG9A in lipid trafficking and mobilization both *in vitro* in human cells and *in vivo* in *C. elegans* [14]. Similar phenotypes have been observed in other autophagy deficiency models, such as liver-specific *Atg5* [21], *Pik3c3* [22], and *Atg7* [24] cKO, and *Atg7* null [23] mice. These studies and ours suggest a general role of autophagy in lipid droplet homeostasis [55] but alternatively, specific functions of ATG9A associated with its intrinsic role in facilitating lipid mobilization between membranes [14]. According to one potential explanation, preventing the flux of phospholipids from the endoplasmic reticulum and other organelles to the autophagosome in *Atg9a-cKO* causes aberrant accumulation of lipid droplets and oxidative stress, which negatively affect mitochondria and cellular homeostasis. However, a decrease in hepatic lipid accumulation in the *Atg7* [56–58] and upstream *Fip200* [59] cKO mice was also reported, offering more than one interpretation of the involvement of autophagy. These studies argue that the reduced formation of lipid droplets in the autophagy cKO models might be due to lower expression of lipogenic genes and reduced fatty acid supply from non-liver sources [57]. Differences in genetic background, age of the animals, and whether mice are fed or starved before tissue collection could lead to different phenotypes. Furthermore, most of the previous reports focused on stages where hepatic tumors were already present, adding to the complex metabolic environment experienced by non-tumor tissue. Additionally, extrapolation of results from autophagy KO cells *in vitro* must be exercised with caution given the complex and dynamic crosstalk between the liver and other organs *in vivo*. This scenario cannot be replicated in a dish – thus the importance of animal studies. Nonetheless, we argue there is still a need to systematically evaluate these differences as well as generate and analyze other ATG-cKO models (and targeted mutants that can disrupt specific structural features) to untangle the role of autophagy in lipid metabolism, given the recent discoveries about the involvement of some players of the Atg8-conjugation machinery in endosomal functions different from canonical autophagy [60].

Our findings also underscore the systemic implications of hepatic ATG9A loss. Elevated cholesterol and reduced albumin levels in blood suggest metabolic stress and early liver dysfunction [61–63] in a sex-dimorphic fashion. While circulating cholesterol levels were increased in both sexes, only ALT levels were significantly increased in males (Figure 6C,E). Although we have not addressed the origins of these differences, it would be of significant interest to design follow-up studies that specifically investigate them, given the widespread knowledge that both major risk factors and the prevalence and severity of certain liver disorders are higher in men than women during reproductive age [64,65]. To our knowledge, this is the first study emphasizing sex differences in an autophagy cKO mouse model.

Proteomics analysis of the liver confirmed disruptions in lipid metabolism and oxidative stress pathways, further linking ATG9A deficiency to impaired detoxification mechanisms (Figure 7). Previous liver autophagy-deficient mice have been shown to exhibit upregulation of similar pathways, highlighting an early common stress response to the absence of autophagy [66].

Our study benefited from a robust conditional knockout model, allowing tissue-specific exploration of ATG9A’s function, unlike previous reports of ATG9A’s embryonic lethality [13,16]. As the study only lasted for 3 months, we could not ascertain the long-term implications of our findings, especially since previous studies in autophagy-deficient livers showed tumorigenesis at advanced ages. Similarly, early time points could narrow down the exact moment when ATG9A becomes critical to the phenotypes observed: does the absence of ATG9A impair proper liver development or is the phenotype due to accumulated liver toxicity like that observed in the KO of downstream autophagy players? Some of these questions could be answered more easily if whole tissue ‘omics (RNAseq, proteomics, and lipidomics) studies were conducted at different developmental times. There is also a need for further investigation into the mechanisms involved in ATG9A’s regulation of lipids. It would be important to focus future studies on ATG9A’s role in mitochondrial-lipid interactions and how it may affect chronic liver disease. Additionally, given the remarkable role played by the liver in controlling whole-body metabolism, these cKO mice could be helpful to study the systemic metabolic effects of other metabolic stressors, such as high fat-high sugar or low-protein diets, starvation, exercise, and endotoxin-induced liver injury, all paradigms used to test autophagy regulation’s limits.

## Materials and methods

### Animals

All animal experiments were performed under protocol #21–021 approved by the NICHD Animal Care and Use Committee, following the NIH Guide for the Care and Use of Laboratory Animals. In groups of no more than four mice per cage, food, and water were provided *ad libitum* and a 12 h light/dark cycle was observed. *Atg9a*^*LoxP/LoxP*^ mice (C57BL/6N ES cell line RENKA) were generated and provided by the team of Dr. Yasuo Uchiyama (described in [16]). *Atg9a*^*LoxP/LoxP*^ mice were crossed with homozygous Albumin-Cre mice (*Alb-Cre*^*tg/tg*^) transgenic mice (provided by Dr. Jake Liang, Liver Diseases Virology Section, NIDDK, NIH and described in [67]) to generate liver-specific *Atg9a* conditional knockout mice carrying a deletion of exons 6 and 7 of the *Atg9a* gene (*Atg9a*-cKO). A single animal is considered the experimental unit in all the experiments. Controls groups have been included in every experiment. There was no randomization, and no animals or experimental units were excluded from the study and analysis. Confounders were not controlled. No *a priori* sample size calculation was performed. We estimated the number of groups and animals based on previous studies of this type.

### Genotyping

During weaning, genomic DNA was isolated from tail biopsies using KAPA Fast genotyping mix (Sigma, MGKITKB). For *Atg9a* genotyping, the following primers were used to distinguish between wild-type (WT) and *Atg9a LoxP* alleles: F: 5’-GGATGATATGTATTCCTGAG; R: 5’-TCCTGACCTGCTGTTCCAGTTCAG. DNA amplification was performed with KAPA Fast genotyping mix and following the amplification protocol: 3 min at 95°C, followed by 30 cycles composed of 15 s at 95°C, 15 s at 60°C and 15 s at 72°C and finished by 2 min at 72°C. The set of primers amplified a 404 bp fragment from the WT allele and a 554 bp fragment from the *Atg9a LoxP* allele. The size of the PCR products was determined on a 1% agarose gel.

For the *Alb-Cre* locus genotyping, the following primers were used to distinguish between WT and Cre alleles: WT_F: 5’-TGCAAACATCACATGCACAC; Cre_F: 5’-GAAGCAGAAGCTTAGGAAGATGG; R: 5’-TTGGCCCCTTACCATAACTG. DNA amplification was performed with KAPA2G Fast Hotstart PCR kit (Sigma, 2GFHSNTMGKB) and following the amplification protocol: 2 min at 94°C, followed by 10 cycles composed of 20 s at 94°C, 15 s at 65°C, and 10 s at 68°C, followed by 28 cycles composed of 15 s at 94°C, 15 s at 60°C, and 15 s at 72°C, and finished by 2 min at 72°C. The set of primers amplified a 351 bp fragment from the WT allele and a 390 bp fragment from the Cre allele.

### Biochemical analysis

Whole blood samples were centrifuged for 10 min at 3,000 rpm for 10 min at 4°C. Serum was separated from red blood cells and stored at 4°C. Samples were analyzed with a Roche Cobas 6000 for triglycerides (TAG), glucose, cholesterol, alanine aminotransferase (ALT), alkaline phosphatase (ALP), and albumin. The degree of hemolysis was measured, and a threshold was set to each analysis to accept the sample: hemolysis index up to 700 for TAG and cholesterol, hemolysis index up to 1000 for glucose and albumin, and hemolysis index up to 200 for ALT and ALP.

### Histology

Livers were harvested and fixed for 24 h by immersion in 4% PFA at 4°C followed by storage in PBS. Paraffin embedding, sectioning, hematoxylin and eosin (H&E) staining and Oil Red O staining of control and *Atg9a*-cKO livers were performed by Histoserv, Inc. (Germantown, MD). Analyses by pathologists were blinded (for both sex and genotype in all the samples provided) during the assessment of the histological sections.

### BrdU and TUNEL assays

Mouse tissues were fixed for FFPE and sectioned at 4–5 µm thickness. For BrdU staining, BrdU incorporation was performed post-fixation *in vitro*. Sections were first treated with 3% hydrogen peroxide for 10 min to block endogenous peroxidase activity. Sections were then incubated with a primary anti-BrdU antibody (Invitrogen, MA3–071), followed by a biotinylated secondary antibody (Invitrogen, 62–6540) and avidin-biotin complex (ABC) reagent, and detection was performed using a chromogen, with hematoxylin counterstaining (Thermo Scientific 32,050). TUNEL staining was performed by permeabilization of sections with proteinase K, incubation with Terminal deoxynucleotidyl Transferase (TdT) enzyme and labeled dUTPs (Sigma-Aldrich, QIA33), and visualization with a chromogen, followed by hematoxylin counterstaining. Stained sections were examined under a brightfield optical microscope, and apoptotic cells were identified based on BrdU or TUNEL positivity, with quantification performed in randomly selected fields.

### Immunohistochemistry of liver sections

Livers were harvested and fixed for 24 h by immersion in 4% PFA at 4°C followed by storage in PBS. Subsequently, the livers were transferred to 30% sucrose in 0.1 M PBS overnight before mounting in OCT compound (Sakura, 4583). Sagittal free-floating sections were prepared on a Leica 9000s microtome and stored at −80°C. A circle was drawn around each tissue section with a Pap pen (Thermo Fisher Scientific, R3777). Sections were rinsed in PBS and subsequently blocked with 5% normal goat serum in PBS containing 0.3% v/v Triton X-100 and 0.5% BSA (blocking solution) at room temperature (RT) for 1 h. Primary antibodies were diluted in 1% normal goat serum in PBS containing 0.3% v/v Triton X-100 and 0.5% BSA (carrier solution) and the sections were incubated overnight at 4°C. After washing three times with carrier solution, sections were incubated for 1 h at RT with appropriate fluorophore-conjugated secondary antibodies diluted in carrier solution. After three washes with carrier solution, sections were mounted on coverslips with DAPI-containing mounting medium (Electron Microscopy Sciences 17,984–24). Confocal microscopy images were collected using an Olympus confocal microscope with a Plan Apochromat 63x objective.

### Antibodies and fluorescent probes

We used the following primary antibodies: anti-ATG7 (rabbit monoclonal, Cell Signaling, 8558), anti-ATG9A (rabbit monoclonal, Cell Signaling 13,509), anti-LC3B (rabbit monoclonal, Cell Signaling, 3868), anti-NQO1 (rabbit monoclonal, Abcam, ab80588), anti-PLIN3 (mouse polyclona, Proteintech 10,694–1-AP), and anti-SQSTM1/p62 (guinea pig polyclonal, Progen, GP62-C). We also used the following secondary antibodies: Alexa Fluor 488-conjugated donkey anti-rabbit IgG (Invitrogen, A21206), HRP-conjugated anti-actin (Sigma, A3854), HRP-conjugated donkey anti-rabbit IgG (GE Healthcare, NA934V), and HRP-conjugated donkey anti-guinea pig IgG (Jackson Immuno Research, 706–035–148). BODIPY 493/503 (referred to as BODIPY 493) (Thermo Fisher Scientific, D3922) was used as a fluorescent lipid probe.

### Image analysis

Images were analyzed using the “Analyze Particles” function in ImageJ (NIH, USA). For each analyzed image, individual cell boundaries were manually outlined using the “freehand selection tool” to define Regions of Interest (ROI), representing each cell area. For each channel, manual thresholding was applied to segment the fluorescent signal. Particle measurements were then performed within each ROI using size and circularity default parameters within the limit to threshold. Data was plotted as quantification of particles (puncta number and size) per cell and per area.

### SDS-PAGE and immunoblotting

Livers were mechanically homogenized in 10 mM HEPES pH 7.5, 150 mM NaCl, 1 mM EDTA, supplemented with protease inhibitors (Millipore Sigma, COEDTAF-RO) using a glass crusher. After homogenization, 1% v/v Triton X-100 was added, samples were incubated on ice for 30 min and centrifuged at 16,000 ×g for 10 min. The supernatant was transferred to a fresh tube. Alternatively, primary hepatocytes in culture were scraped from the plate in PBS 1x, and centrifuged at 1,000 g for 5 min. The pelleted cells were resuspended in 10 mM HEPES pH 7.5, 150 mM NaCl, 1 mM EDTA, 1% v/v Triton X-100 supplemented with protease inhibitors, incubated on ice for 30 min and centrifuged at 16,000 ×g for 10 min. Total protein concentration was estimated using a modified Bradford method (Bio-Rad 5,000,002). Samples were then denatured in sample buffer (Bio-Rad 1,610,747) containing 2.5% v/v 2-mercaptoethanol (Millipore Sigma, M6250) for 5 min at 37°C, resolved by SDS-PAGE, and transferred onto a nitrocellulose membrane (Bio-Rad 1,620,215). Molecular markers were used in every experiment (Thermo Fisher Scientific 26,620). Membranes were blocked using 3% blotting-grade blocker (Bio-Rad 1,706,404) in PBS + 0.1% Tween 20 (Sigma-Aldrich, P1379) for 2 h at 4°C. Primary antibodies were then incubated overnight at 4°C. After washing the membranes, HRP-conjugated secondary antibodies were used at room temperature for 1 h. Clarity (Bio-Rad 1,705,060) or West Femto ECL kit (Thermo Fisher Scientific 34,094) were used to develop the immunoblots using a ChemiDoc system (Bio-Rad).

### Isolation and culture of primary hepatocytes

Mouse primary hepatocytes were purified and cultured following the protocol published by Charni-Natan and Goldstein [68] without modifications.

### Transmission electron microscopy

Small liver samples (less than 1 mm^3^) were fixed for 1.5 h in 2% (w/v) formaldehyde and 2% (w/v) glutaraldehyde in 0.1 M sodium cacodylate (pH 7.4), postfixed in 0.5% reduced OsO_4_, (0.5% OsO_4_/0.5% potassium ferrocyanide in the same buffer) and stained *en bloc* with 2% (w/v) aqueous uranyl acetate. Samples were further washed and dehydrated with series of ethanol concentrations, penetrated with EMbed 812 (Electron Microscopy Sciences 14,120) and placed in flat molds. The resin was subsequently polymerized at 65°C for 60 h. Thin 70-nm sections were cut using a Leica EM UC7 microtome and subsequently were post-stained with lead citrate. We examined the samples using a FEI Tecnai 20 transmission electron microscope operating at 120 kV and recorded the images with an AMT XR81CCD camera.

### Isolation of RNA and gene expression analysis

RNA was purified from FFPE sections using the Maxwell RSC RNA FFPE Kit (Promega, AS1440) with the Maxwell RSC Instrument, following the manufacturer’s protocol. Briefly, 3–4 sections (~30 μm thickness) were combined in a single nuclease-free tube for RNA extraction. The initial sections from the surface were discarded. Excess paraffin was removed using a scalpel. RNA was eluted in 50 μL of RNase-free water, and its concentration was determined using a Nanodrop spectrophotometer. A total of 100 ng of RNA was reverse transcribed into cDNA using the SuperScript^TM^ III First-Strand Synthesis System (Invitrogen 18,080,051) according to the manufacturer’s instructions, with a mix of oligo(dT) and random hexamer primers. The following primers were designed (NCBI Primer-BLAST tool) to amplify short amplicons of 60–85 bp: *Nlrp3*_F: 5’-TGGCTGTGTGGATCTTTGCT, *Nlrp3*_R: 5’-ACGTGTCATTCCACTCTGGC; *Il33*_F: 5’-ATCCAAGCATTTGCTGCGTC, *Il33*_R: 5’-GGAGGCAGGAGACTGTGTTA. *Gapdh* was used as a housekeeping gene: *Gapdh*_F: TTCACCACCATGGAGAAGGC, *Gapdh*_R: GGCGGAGATGATGACCCTTT. Quantitative PCR (qPCR) was performed using PowerUp SYBR Green Master Mix (Thermo Fisher, A25742).

### Mass spectroscopy

A 10 mM solution of tris(2-carboxyethyl)phosphine (TCEP) (Sigma Aldrich 75,259) was used to reduce in-gel samples, followed by alkylation using a 10 mM solution of N-ethylmaleimide (NEM) (Sigma Aldrich 04,259). A 1:20 (w/w) ratio of trypsin (Promega, V5280) to sample was used at 37°C overnight for digestion. After extraction, peptides were desalted using Oasis HLB plates (Waters). For data acquisition, an Orbitrap Lumos mass spectrometer (Thermo Scientific) was coupled to an Ultimate 3000 HPLC (Thermo Scientific). An ES911 column was used to separate peptides using a gradient with mobile phase B (0.1% formic acid in LC-MS grade acetonitrile) increased from 3 to 20% in 83 min, from 20 to 30% in 12 min, at a flow rate of 300 nL/min. LC-MS/MS data were acquired in data-dependent mode. A survey scan was conducted at a resolution of 120k and a mass range of 400 to 1500 m/z. The MS1 cycle time was set to 3 s. As many MS2 scans as possible were acquired within the cycle time. With an isolation window of 1.6 m/z, MS2 data were acquired using an ion trap with rapid scan rates. Peptide fragmentation was achieved using the CID method with a collision energy fixed at 30. For MS2 scan to be triggered, a signal intensity of 1x10^4^ was required.

Proteome Discoverer 2.4 Software was used to process raw data. The data were searched against the Sprot Mouse database. For the precursor and fragment, mass tolerances were set at 10 ppm and 0.6 Da, respectively. A maximum of two missed cleavages was allowed. NEM on cysteines was set as a fixed modification. Other modifications included Oxidation (M), Met-loss (Protein N-term) and Acetylation (Protein N-term). Based on the abundance of the unique peptides matched to each protein, protein abundances were calculated. Normalization was performed against the total amount of peptides. Each group’s median protein abundance was used to calculate protein ratios. Low Abundance Resampling was used to impute missing values. Hypothesis tests were conducted using the ANOVA method (Individual Proteins).

## Supplementary Material

Supplemental Material

## Data Availability

MS raw data was deposited in the public MassIVE database under identifier MSV000097095. Supplementary data can be accessed on Mendeley Data (10.17632/hpmzydbkyp.1). Any other data supporting the findings of the current study are available from the corresponding author upon request.

## Abbreviations

ALP: alkaline phosphatase
ALT: alanine transaminase
ATG: autophagy related
BODIPY: boron dipyrromethene
BSA: bovine serum albumin
cKO: conditional knockout
CTL: control
DAPI: 4,’6-diamidino-2-phenylindole
dsDNA: double-stranded DNA
dUTP: deoxyuridine triphosphate
ECL: enhanced chemiluminescence
EDTA: Ethylenediaminetetraacetic acid
FFPE: formalin fixed paraffine embedded
H&E: hematoxylin and eosin
HEPES: 2-[4-(2-hydroxyethyl)piperazin-1-yl]ethanesulfonic acid
HRP: horseradish peroxidase
KO: knockout
LC-MS: liquid chromatography-mass spectrometry
NEM: N-ethylmaleimide
OCT: optimal cutting temperature
ORO: oil red O
PBS: phosphate-buffered saline
PCR: polymerase chain reaction
RNAseq: RNA sequencing
RT: room temperature
SDS-PAGE: sodium dodecyl sulfate-polyacrylamide gel electrophoresis
TCEP: tris(2-carboxyethyl)phosphine hydrochloride
TdT: terminal deoxy-nucleotidyl transferase
TUNEL: terminal deoxy-nucleotidyl transferase-mediated dUTP nick end-labeling
WT: wild-type
