# Supplementary figures and images for: Liver-specific loss of Atg9a perturbs lipid metabolism and hepatocyte integrity
Source: Autophagy Rep. 2025 Sep 2;4(1):2551028. doi: 10.1080/27694127.2025.2551028 (PMC12408054; doi:10.1080/27694127.2025.2551028)

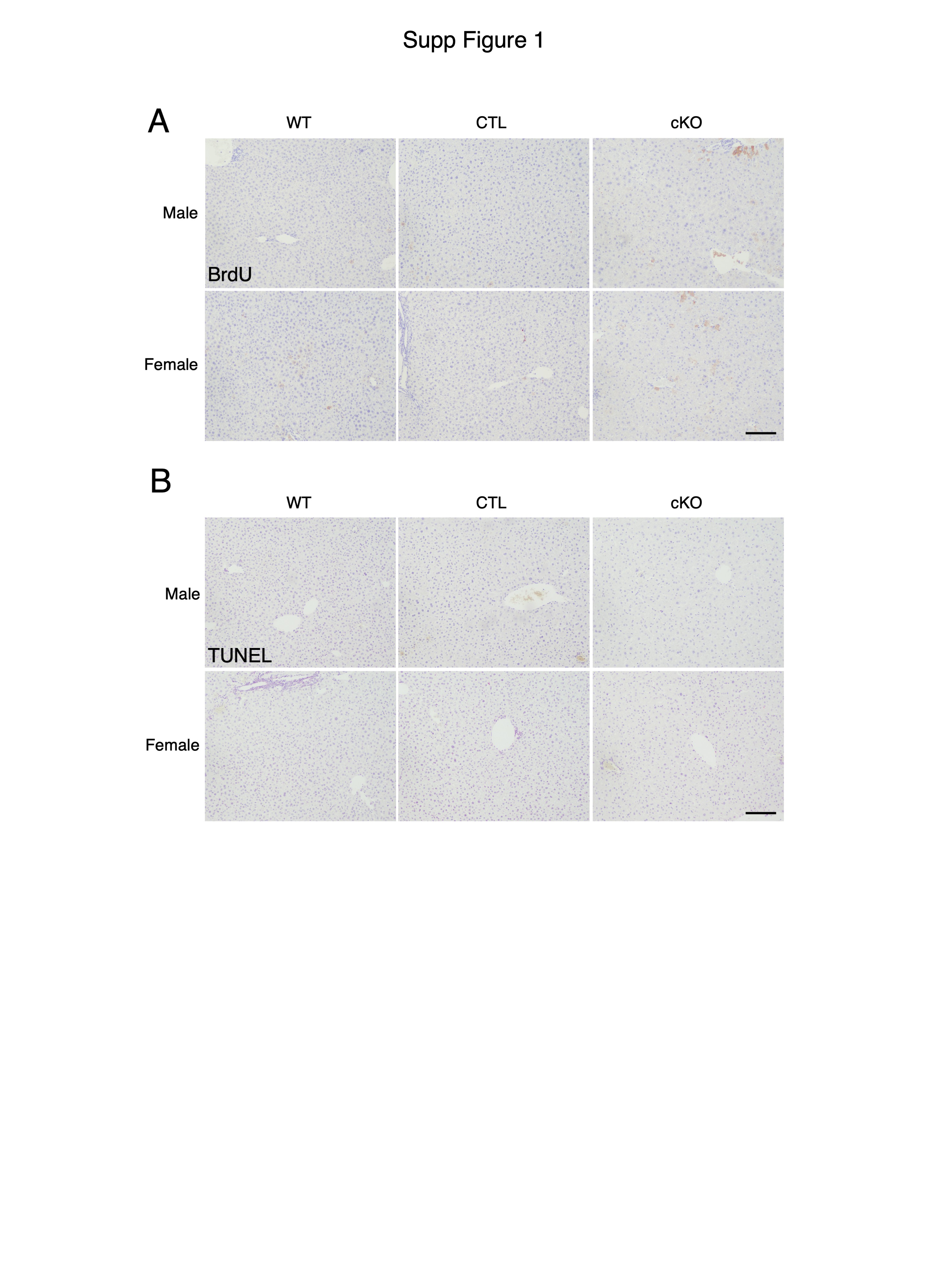

Supplement: Supplemental Material [file KAUO_A_2551028_SM4132.zip › SuppFig1.jpeg]
